# miR‐642a‐5p partially mediates the effects of lipopolysaccharide on human pulmonary microvascular endothelial cells via eEF2

**DOI:** 10.1002/2211-5463.12969

**Published:** 2020-10-16

**Authors:** Liming Fei, Gengyun Sun, Qinghai You

**Affiliations:** ^1^ Department of Respiratory and Critical Care Medicine The First Affiliated Hospital of Anhui Medical University Hefei China

**Keywords:** acute lung injury and acute respiratory distress syndrome, eukaryotic elongation factor 2, lipopolysaccharide, miR‐642a‐5p, PMVECs

## Abstract

Inhalation or systemic administration of lipopolysaccharide (LPS) can induce acute pulmonary inflammation and lung injury. The pulmonary vasculature is composed of pulmonary microvascular endothelial cells (PMVECs), which form a semiselective membrane for gas exchange. The miRNA miR‐642a‐5p has previously been reported to be up‐regulated in patients with acute respiratory distress syndrome; thus, here, we examined whether this miRNA is involved in the effects of LPS on PMVECs. The levels of miR‐642a‐5p and mRNA encoding eukaryotic elongation factor 2 (eEF2) were detected by quantitative RT‐PCR. Moesin and eEF2 protein levels were tested by western blot assay. Dual‐luciferase reporter assay was used to examine the relationship between miR‐642a‐5p and eEF2. Cell viability was assessed using the 3‐(4,5‐dimethylthiazol‐2‐yl)‐2,5‐diphenyltetrazolium bromide assay, and cell permeability was analyzed using the transendothelial electrical resistance assay. We report that miR‐642a‐5p levels are significantly up‐regulated in LPS‐stimulated PMVECs, and miR‐642a‐5p contributes to LPS‐induced hyperpermeability and apoptosis of PMVECs. LPS treatment results in down‐regulation of eEF2 in PMVECs. Overexpression of eEF2, a direct target of miR‐642a‐5p, inhibited the effect of LPS on PMVECs. miR‐642a‐5p promoted LPS‐induced hyperpermeability and apoptosis by targeting eEF2. Thus, miR‐642a‐5p and eEF2 may serve as potential targets for acute lung injury/acute respiratory distress syndrome diagnosis or treatment.

## Graphical abstract

miR‐642a‐5p promotes lipopolysaccharide‐induced hyperpermeability and apoptosis by targeting eukaryotic elongation factor 2. Thus, miR‐642a‐5p and eukaryotic elongation factor 2 may be potential targets for acute lung injury/acute respiratory distress syndrome diagnosis or treatment.

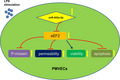

**Abbreviations**
ALI: acute lung injury
ARDS: acute respiratory distress syndrome
eEF2: eukaryotic elongation factor 2
LPS: lipopolysaccharide
MTT: 3‐(4,5‐dimethylthiazol‐2‐yl)‐2,5‐diphenyltetrazolium bromide
MUT‐eEF2: mutated eEF2
NC: negative control
NLRP3: NOD‐like receptor protein 3
PMVEC: pulmonary microvascular endothelial cell
qRT‐PCR: quantitative RT‐PCR
SD: standard deviation
TER: transendothelial electrical resistance
TLR4: Toll‐like receptor 4
WT: wild‐type

Acute lung injury (ALI) and acute respiratory distress syndrome (ARDS) are severe lung conditions, often accompanied by pulmonary microvascular leakage, pulmonary edema and pleural effusions, leading to high morbidity and mortality [1, 2]. It has been reported that pathogenic infection, such as sepsis, is the main cause of ALI/ARDS [3]. Usually, the pulmonary vasculature composed of pulmonary microvascular endothelial cells (PMVECs) forms a semiselective membrane, which is crucial for gas exchange and preventing fluid leakage. However, when exposed to agonists, such as lipopolysaccharide (LPS), cytokines or chemokines, the endothelial cells become activated. The activation of PMVECs results in enhanced permeability, increased leukocyte adhesion and promotion of a shift from the hemostatic balance to a procoagulant phenotype [4, 5]. These changes allow increased blood flow to the insulted area, aiming to sequester the infection and repair the injury. However, dysregulation of the PMVEC activation disturbs the homeostatic state, leading to diffuse damage of endothelial cells and destruction of endothelial cell barriers [6, 7]. In sepsis, endothelial activation and dysfunction are critical determinations of the host response. Therefore, a better understanding of the mechanism for elucidating the high permeability of endothelial cells is essential, which may provide new ideas for future therapeutic treatment development.

LPS is an endotoxin released by Gram‐negative bacteria [8]. Its primary pathogenic mechanism is to activate endothelial cells. Activation and dysfunction of the PMVECs are characterized by changes in cell function and structural integrity, production of more cytokines and chemokines, increased permeability and vasodilation. Those changes contribute to the development of pulmonary edema and ARDS [9, 10, 11]. Thus, it is of great importance to explore the molecular mechanisms underlying LPS‐induced permeability change of PMVECs.

miRNAs are small noncoding RNAs that inhibit the expression of target genes by recognizing or degrading mRNA at specific sites on the target genes. miRNAs play critical roles in the regulation of various biological processes, including cell proliferation, cycle, apoptosis, angiogenesis, inflammatory response and infection [12, 13]. miR‐642a‐5p was reported to be abnormally expressed in multiple human cancers, including lung cancer, colon cancer, pediatric embryonic central nervous system tumor, myeloma and cervical cancer [14, 15, 16, 17, 18]. Despite that the expression profile and biological function roles of miR‐642a‐5p in some types of human cancer have been revealed, whether miR‐642a‐5p is involved in ALI/ARDS is unknown. A recent publication indicated that the whole blood levels of miR‐642a‐5p are up‐regulated in patients with ARDS and may serve as a risk factor for ARDS [19]. This exciting report encouraged us to pursue the biological function role of miR‐642a‐5p in LPS‐stimulated pulmonary endothelial cells. Therefore, this study focused on exploring the roles of miR‐642a‐5p in LPS‐induced PMVECs permeability and viability.

## Materials and methods

### Cell culture

Human PMVECs were obtained from American Type Culture Collection (Manassas, VA, USA). PMVECs were cultured in Dulbecco's modified Eagle's medium with 10% fetal bovine serum (Gibco, Carlsbad, CA, USA). Cells were incubated in a 5% CO_2_ humidified atmosphere at 37 °C.

### Quantitative RT‐PCR

Total RNA was extracted from cells by using TRIzol reagent (Invitrogen, Carlsbad, CA, USA). Quantitative RT‐PCR (qRT‐PCR) was performed using StepOnePlus Real‐Time PCR System (Applied Biosystems, Waltham, MA, USA) according to the manufacturer's instructions. Five micrograms of total RNA was reversely transcribed to cDNA by using First Strand cDNA Synthesis Kit (Applied Biosystems) with specific reverse transcriptase primers for miRNAs. The qRT‐PCR analyses were performed using the standard SYBR Green qPCR Mix. All reactions were done in triplicate. The $2^{‐\Delta \Delta C_{t}}$ method was used to calculate relative quantities. miRNA expression is normalized to U6 snRNA. The primers used in this article are shown as follows: miR‐642a‐5p, RT GTCGTATCCAGTGCAGGGTCCGAGGTATTCGCACTGGATACGACCAAGAC, forward GCGGTCCCTCTCCAAATGT, and reverse AGTGCAGGGTCCGAGGTATT; U6 (internal control gene), RT GTCGTATCCAGTGCAGGGTCCGAGGTATTCGCACTGGATACGACAAAATATGGAA, forward CTCGCTTCGGCAGCACA, and reverse AACGCTTCACGAATTTGCGT; eukaryotic elongation factor 2 (eEF2), forward CCATCTCCCTCTTCTACGA, and reverse GCGGTCCATCTTGTTCAT; glyceraldehyde‐3 phosphate dehydrogenase (internal control gene) primer, forward ACAACTTTGGTATCGTGGAAGG, and reverse GCCATCACGCCACAGTTTC.

### Western blot

Total proteins were extracted from cells using radioimmunoprecipitation lysis buffer supplemented with the protease inhibitor cocktail. An equal amount of proteins (50 µg) was loaded and separated by 10% SDS/PAGE. The proteins were transferred onto a poly(vinylidene fluoride) membrane (Millipore, Billerica, MA, USA). The membranes were blocked by 5% BSA in Tris‐buffered saline and Tween 20, and incubated overnight at 4 °C with primary antibodies. Then the membranes were washed and incubated with horseradish peroxidase‐conjugated secondary antibodies. The target proteins were assessed using an electrochemiluminescence system. The targeted protein band's signal was quantified using image j software (National Institutes of Health, Bethesda, MD, USA).

### Dual‐luciferase reporter assay

To construct luciferase reporter vectors, we synthesized and subcloned the cDNA fragments containing the wild‐type (WT) eEF2 (WT‐eEF2) or the mutated eEF2 (MUT‐eEF2) into the downstream of the luciferase gene in the psi‐CHECK2 vector. WT‐eEF2 or MUT‐eEF2 and miR‐642a‐5p mimics or negative controls (NCs) were cotransfected into PMVECs using Lipofectamine 3000 according to the manufacturer's instructions. Luciferase activities were examined using the dual‐luciferase reporter assay system (Promega, Madison, MI, USA).

### Transendothelial electrical resistance assay

The transendothelial electrical resistance (TER) of PMVEC monolayers was measured using an STX2 electrode and EVOM2 meter according to the manufacturer's instructions (World Precision Instruments, Sarasota, FL, USA). PMVECs were seeded on fibronectin‐coated transwell filters at 100% confluence. After LPS treatment, the resistance values of each sample were measured sequentially. The resistance of a blank control (culture insert without cells) was also measured. The mean resistant value was expressed in the unit (Ω × cm^2^) after subtraction of the blank control.

### 3‐(4,5‐Dimethylthiazol‐2‐yl)‐2,5‐diphenyltetrazolium bromide assay

After LPS treatment, the medium was carefully discarded from cell cultures. Fifty microliters of fresh media and 50 µL of 3‐(4,5‐dimethylthiazol‐2‐yl)‐2,5‐diphenyltetrazolium bromide (MTT) solution were added into each well, and the plate was incubated for 3 h at 37 °C. A total of 150 µL of MTT solvent was then added into each well after incubation, and absorbance of each well at 590 nm (*A*_590 nm_) was read using a plate reader.

### Caspase‐3 activity measurement

Caspase‐3/CPP32 Fluorometric assay kit (Biovision, San Francisco, CA, USA) was used to measure the caspase‐3 activity. In brief, cells were lysed in 50 µL cell lysis buffer. Fifty microliter of reaction buffer and 5 µL of DEVD‐AFC substrate (1 mm) were added into each sample. After incubation for 2 h in 37 °C, samples were read in a fluorometer equipped with a 400‐nm excitation filter and 505‐nm emission filter.

### Annexin V and propidium iodide staining

The Annexin V and propidium iodide staining kit was purchased from BD Biosciences (San Jose, CA, USA). Cells were washed twice with cold PBS and resuspended in binding buffer (100 µL). Annexin V (2 µL) and propidium iodide (2 µL) were added into the cell resuspension and incubated for 15 min at 4 °C in the dark. Cells were further washed twice, resuspended in binding buffer (400 µL) and analyzed by flow cytometry (Accuri C6; BD Biosciences).

### Statistics

Statistical data were analyzed by spss statistical software 20 (SPSS, Chicago, IL, USA) for Windows. Data are presented as mean ± standard deviation (SD). Student's *t*‐tests or one‐ or two‐way ANOVA was performed to determine the statistically significant differences among different experimental groups. A *P* value <0.05 was considered statistically significant.

## Results

### LPS induces hyperpermeability and apoptosis of PMVECs

**Fig. 1:**
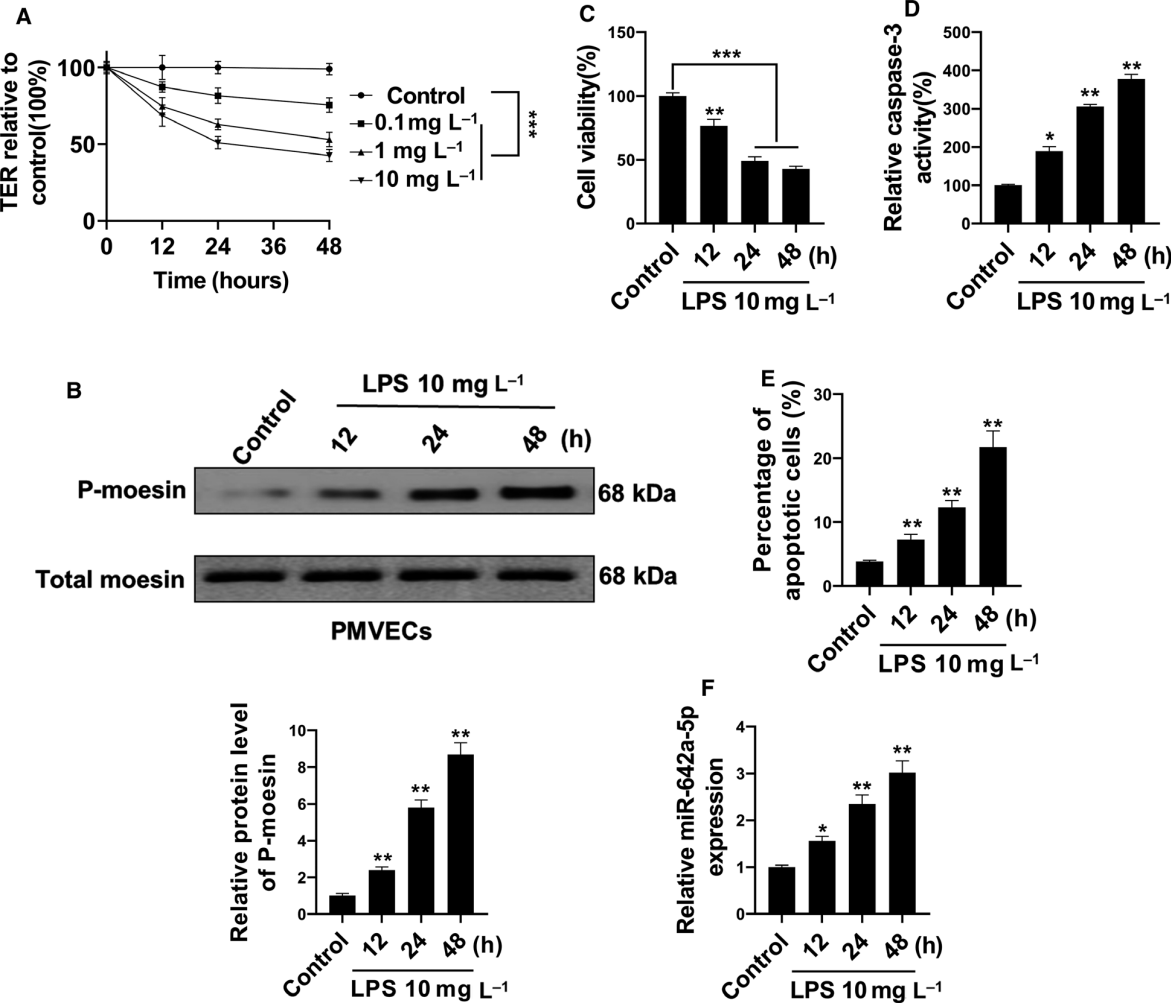
miR‐642a‐5p was induced by LPS stimulation in PMVECs. (A) Cultured human PMVECs grown to confluence on gold electrodes were treated with recombinant LPS (0.1, 1 or 10 mg·L^−1^), and changes in TER were measured at 12, 24 or 48 h. (B) Time‐dependent changes of phosphorylation of moesin in human PMVECs induced by 10 mg·L^−1^ LPS were detected by western blot. (C) The cell viability of human PMVECs induced with 10 mg·L^−1^ LPS for 12, 24 or 48 h was measured by MTT. (D, E) The apoptosis of human PMVECs treated with 10 mg·L^−1^ LPS for 12, 24 or 48 h was measured using the caspase‐3 activity assay and flow cytometry analysis. (F) The level of miR‐642a‐5p in human PMVECs treated with 10 mg·L^−1^ LPS for 12, 24 or 48 h was determined by qRT‐PCR. Two‐way ANOVA and a Bonferroni *post hoc* test (A), or one‐way ANOVA and a Dunn's *post hoc* test were used (B–F). Experiments were repeated independently in triplicate. Data were presented as mean ± SD. *P < 0.05, **P < 0.01, ***P < 0.001 versus control.

To test the effect of LPS on the permeability of barrier‐forming PMVECs, we applied TER assay. PMVECs were treated with LPS (0.1, 1 or 10 mg·L^−1^), and the TER was measured at 12, 24 and 48 h posttreatment. We found that the TER was decreased in a dose‐ and time‐dependent manner (Fig. 1A). Moesin was reported to be involved in LPS‐stimulated cell membrane permeabilization [20]. Indeed, we observed that the expression levels of p‐moesin, but not total moesin, were sharply up‐regulated after LPS treatment (Fig. 1B). We further found that LPS stimulation induced the up‐regulation of the activity of caspase‐3 (Fig. 1D), a crucial mediator of apoptosis, as well as apoptosis (Fig. 1E), in a time‐dependent manner. Concurrently, gradual down‐regulation of PMVECs viability was also observed after LPS treatment (Fig. 1C). These results suggested that decreased PMVECs viability may be responsible for the LPS stimulation‐induced hyperpermeability of PMVEC‐formed barrier. Interestingly, we also found that the expression levels of miR‐642a‐5p also increased in a time‐dependent manner in PMVECs with LPS stimulation (Fig. 1F).

### miR‐642a‐5p in LPS‐stimulated PMVECs

**Fig. 2:**
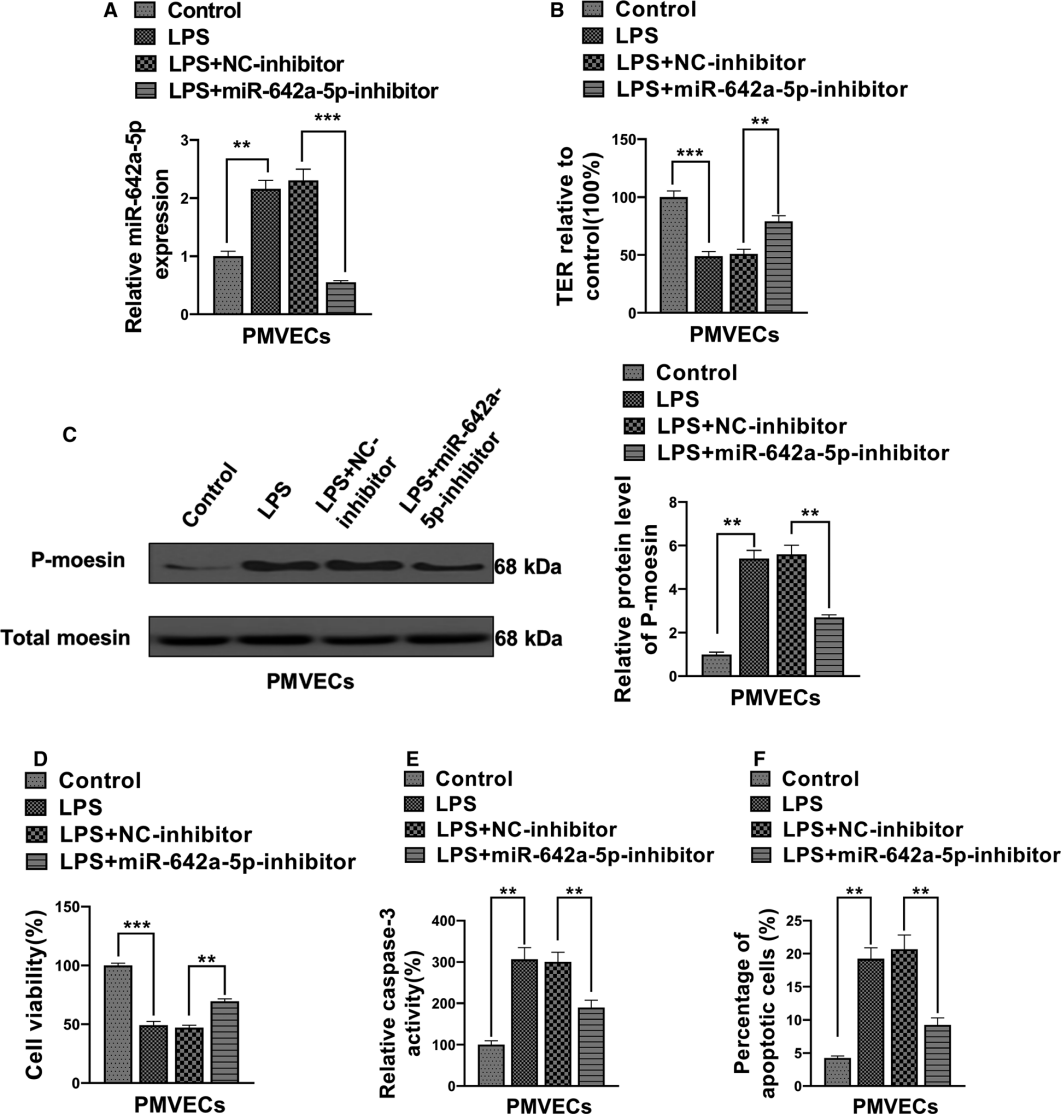
miR‐642a‐5p is involved in LPS‐induced hyperpermeability and apoptosis of PMVECs. (A) The level of miR‐642a‐5p in PMVECs transfected with NC inhibitor or miR‐642a‐5p inhibitor and stimulated by LPS (10 mg·L^−1^, 24 h) was determined by qRT‐PCR. (B) The TER of these PMVECs transfected with NC inhibitor or miR‐642a‐5p inhibitor and stimulated by LPS (10 mg·L^−1^, 24 h) was measured. (C) The phosphorylation level of moesin in these PMVECs transfected with NC inhibitor or miR‐642a‐5p inhibitor and stimulated by LPS (10 mg·L^−1^, 24 h) was measured by western blot. (D) The cell viability of these PMVECs transfected with NC inhibitor or miR‐642a‐5p inhibitor and stimulated by LPS (10 mg·L^−1^, 24 h) was detected by MTT. (E, F) The apoptosis of these PMVECs transfected with NC inhibitor or miR‐642a‐5p inhibitor and stimulated by LPS (10 mg·L^−1^, 24 h) was assessed using the caspase‐3 activity assay and flow cytometry analysis. One‐way ANOVA and a Dunn's *post hoc* test were used. Experiments were repeated independently in triplicate. Data were presented as mean ± SD. **P < 0.01, ***P < 0.001.

To investigate the role of miR‐642a‐5p in LPS‐stimulated hyperpermeability and apoptosis of PMVECs, we first confirmed that LPS treatment increased the expression levels of miR‐642a‐5p in PMVECs. The enhanced miR‐642a‐5p levels were abolished after transfection of the miR‐642a‐5p‐inhibitor (Fig. 2A). The results in Fig. 2B–F showed that knockdown of miR‐642a‐5p partially blocked LPS‐induced up‐regulation of p‐moesin and caspase‐3 levels in PMVECs (Fig. 2C,E), leading to notably abrogating the effects of LPS‐induced hyperpermeability and apoptosis of PMVECs (Fig. 2B,D,F). These results strongly suggest that miR‐642a‐5p plays a positive role in the promotion of LPS‐induced permeability and apoptosis of PMVECs.

### miR‐642a‐5p targets eEF2

**Fig. 3:**
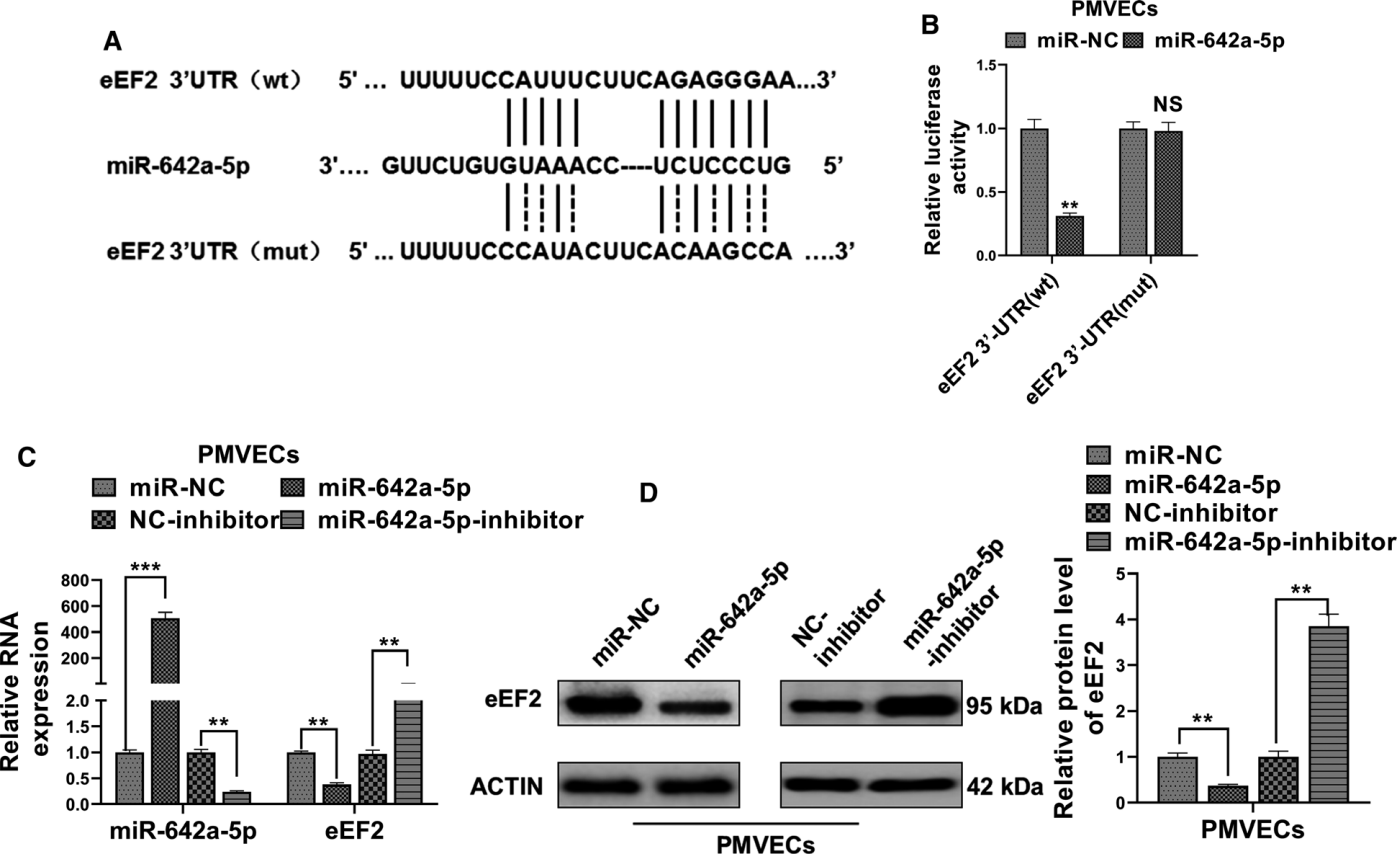
eEF2 is a direct target of miR‐642a‐5p. (A) The prediction for miR‐642a‐5p binding sites on the 3′ UTR of eEF2 and schematic of luciferase reporter vector constructs WT‐eEF2 and the miR‐642a‐5p binding site mutated one (MUT‐eEF2). (B) Relative luciferase activities of psi‐CHECK2 carrying WT‐eEF2 or MUT‐eEF2 in PMVECs cotransfected with miR‐642a‐5p mimics or miR‐NC. (C) The relative RNA expression of miR‐642a‐5p and eEF2 in PMVECs transfected with miR‐642a‐5p mimics and miR‐642a‐5p inhibitors or their respective NCs were detected by qRT‐PCR. (D) The protein level of eEF2 in PMVECs transfected with miR‐642a‐5p mimics and miR‐642a‐5p inhibitors or their respective NCs was detected by western blot. Student's *t*‐test (B) or one‐way ANOVA and a Dunn's *post hoc* test were used (B–D). Experiments were repeated independently in triplicate. Data were presented as mean ± SD. **P < 0.01, ***P < 0.001. NS, not significant.

To explore the potential target of miR‐642a‐5p in PMVECs, we applied a well‐known miRNA target searching tool, TargetScan. We identified that the 3′ UTRs of the eEF2 gene contain a potential binding site of miR‐642a‐5p (Fig. 3A). To further confirm this finding, we subcloned the fragments of the DNA sequence containing the WT or mutant 3′ UTR of eEF2 gene into the downstream of the luciferase gene within the psi‐CHECK2. Forced expression of miR‐642a‐5p significantly reduced the luciferase activity of the plasmid containing the WT, but not the mutant, 3′ UTRs of eEF2 (Fig. 3B). Furthermore, we found that overexpression of miR‐642a‐5p evidently decreased, whereas knockdown of miR‐642a‐5p dramatically increased, the mRNA and protein levels of eEF2 (Fig. 3C,D). These results suggest that miR‐642a‐5p specifically binds to the 3′ UTRs of eEF2 and regulates the expression levels of eEF2 in PMVECs.

### eEF2 in LPS‐stimulated PMVECs

**Fig. 4:**
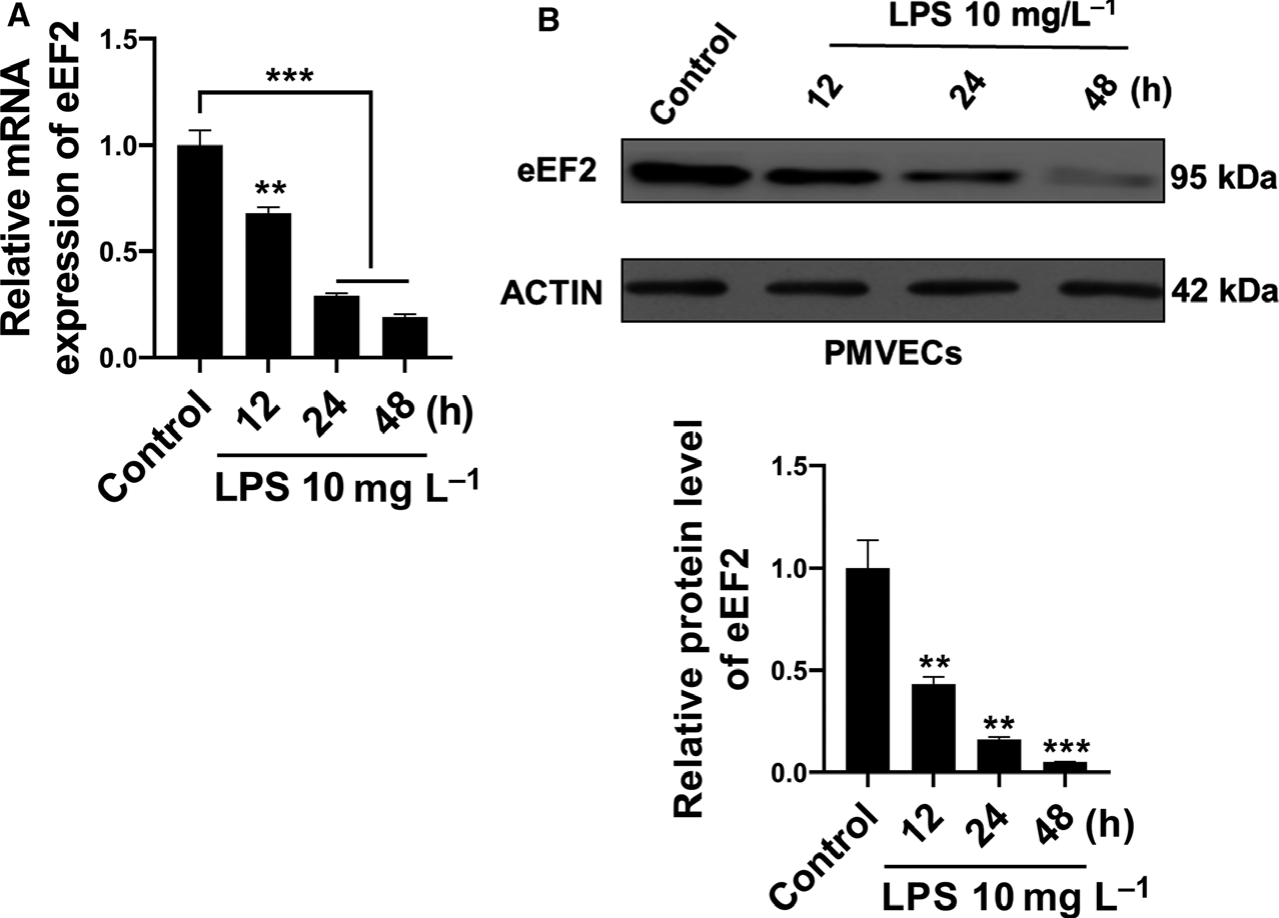
eEF2 was dysregulated in LPS‐induced PMVECs. (A, B) The RNA and protein levels of eEF2 in PMVECs treated with 10 mg·L^−1^ LPS for 12, 24 or 48 h were determined by qRT‐PCR and western blot. One‐way ANOVA and a Dunn's *post hoc* test were used. Experiments were repeated independently in triplicate. Data were presented as mean ± SD. **P < 0.01, ***P < 0.001.

To study whether eEF2 is regulated by LPS in PMVECs, we treated the PMVECs with LPS for 12, 24 and 48 h. In contrast with the changes of miR‐642a‐5p, we found that both the mRNA and the protein levels of eEF2 were significantly decreased in a time‐dependent manner (Fig. 4A,B).

**Fig. 5:**
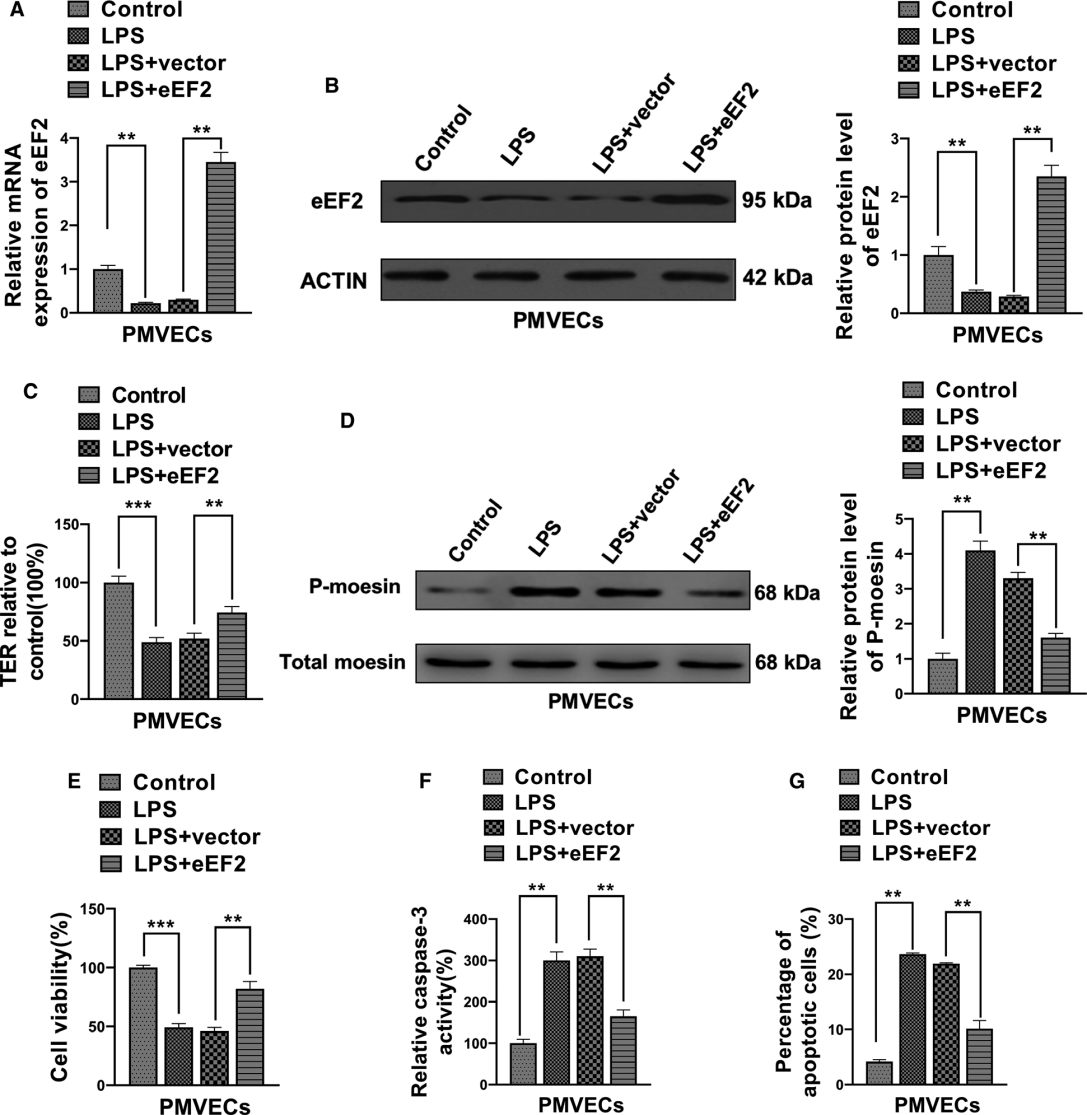
eEF2 inhibited LPS‐induced hyperpermeability and apoptosis of PMVECs. (A, B) The RNA and protein levels of eEF2 in PMVECs transfected with pSin‐eEF2 or empty vector and stimulated by LPS were determined by qRT‐PCR and western blot, respectively. (C) The TER of these PMVECs transfected with pSin‐eEF2 or empty vector and stimulated by LPS was measured by TER assay. (D) The phosphorylation level of moesin in these PMVECs transfected with pSin‐eEF2 or empty vector and stimulated by LPS was measured by western blot. (E) The cell viability of these PMVECs transfected with pSin‐eEF2 or empty vector and stimulated by LPS was measured by MTT. (F, G) The apoptosis of these PMVECs transfected with pSin‐eEF2 or empty vector and stimulated by LPS was measured using the caspase‐3 activity assay and flow cytometry analysis. One‐way ANOVA and a Dunn's *post hoc* test were used. Experiments were repeated independently in triplicate. Data were presented as mean ± SD. **P < 0.01, ***P < 0.001.

To further address the biological relevance of eEF2 on LPS‐mediated permeability and viability of PMVECs, we forced expression of eEF2 on PMVECs. Up‐regulation of the mRNA and protein levels of eEF2 after transfection of eEF2 vector was confirmed by qRT‐PCR and western blot assay, respectively (Fig. 5A,B). Consistent with the results of miR‐642a‐5p knockdown, we found that overexpression of eEF2 partially blocked LPS‐induced up‐regulation of p‐moesin and caspase‐3 activity (Fig. 5D,F), as well as moderately rescued LPS‐induced hyperpermeability and apoptosis of PMVECs (Fig. 5C,E,G).

### miR‐642a‐5p regulates LPS‐induced PMVECs cellular function through targeting eEF2

**Fig. 6:**
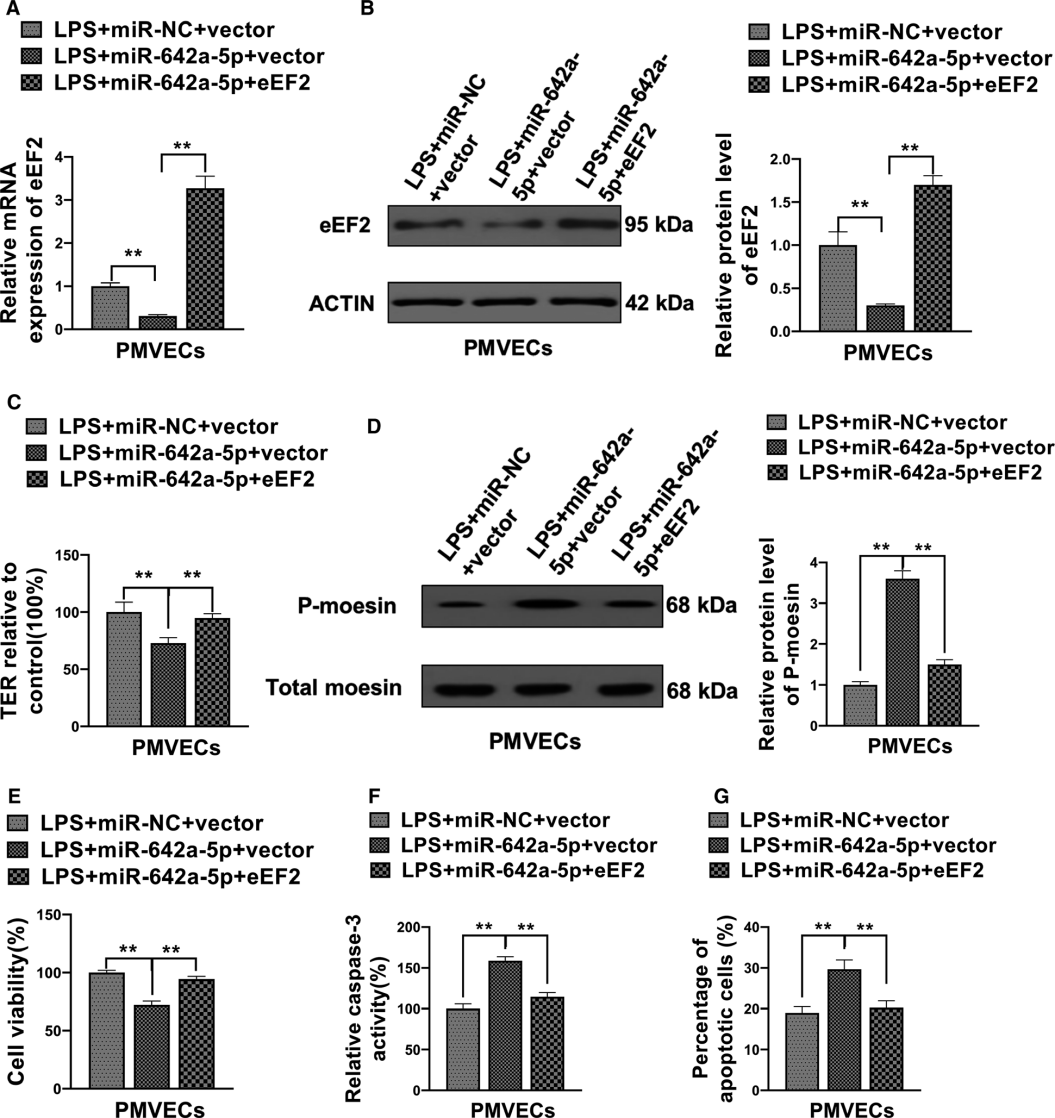
The effect of miR‐642a‐5p on the LPS‐induced hyperpermeability and apoptosis of PMVECs was rescued by eEF2 overexpression. (A, B) PMVECs cotransfected with mimics NC and empty vector (miR‐NC+vector), miR‐642a‐5p mimics and empty vector (miR‐642a‐5p+vector) or miR‐642a‐5p mimics and eEF2 overexpression plasmid (miR‐642a‐5p+eEF2), and then stimulated by LPS. The RNA and protein levels of eEF2 were measured by qRT‐PCR and western blot. (C) The TER of these cotransfected and LPS‐stimulated PMVECs was assessed by TER assay. (D) The phosphorylation level of moesin in these cotransfected and LPS‐stimulated PMVECs was determined by western blot. (E) The cell viability of these cotransfected and LPS‐stimulated PMVECs was detected by MTT. (F, G) The apoptosis of these cotransfected and LPS‐stimulated PMVECs was measured using the caspase‐3 activity assay and flow cytometry analysis. One‐way ANOVA and a Dunn's *post hoc* test were used. Experiments were repeated independently in triplicate. Data were presented as mean ± SD. **P < 0.01.

We have demonstrated that both miR‐642a‐5p and eEF2 participated in LPS‐mediated permeability and viability of PMVECs, and eEF2 is a direct target of miR‐642a‐5p. To further elucidate whether miR‐642a‐5p regulates the biological function of LPS‐stimulated PMVECs through targeting eEF2, we restored the eEF2 expression in miR‐642a‐5p‐overexpressed cells. Figure 6A,B showed that up‐regulation of miR‐642a‐5p markedly decreased mRNA and protein levels of eEF2, and eEF2 plasmid transfection rescued eEF2 expression in miR‐642a‐5p‐overexpressed cells. We observed that the up‐regulation of miR‐642a‐5p in LPS‐treated PMVECs further enhanced p‐moesin levels and caspase‐3 activity (Fig. 6D,F), resulting in a higher apoptosis and permeability rate of PMVECs compared with the miRNA‐NC (control) transfection group. Importantly, the promotion effect of miR‐642a‐5p can be partially blocked by the restoration of eEF2 expression in LPS‐stimulated PMVECs (Fig. 6C,E,G). Collectively, these results suggest that miR‐642a‐5p regulates LPS‐treated permeability and viability of PMVECs through targeting eEF2.

## Discussion

ALIs, including ARDS, are complex inflammatory lung diseases, characterized by intense inflammation in the lung, progressive hypoxemia and reduced lung compliance [21, 22]. It is difficult to accurately assess the incidence of ALI/ARDS worldwide because of geographical variation, inadequate documentation and etiologic variations. It is estimated that there are approximately 200 000 new ALI cases per year with a mortality rate of 40% [23]. The pathogenesis of ALI/ARDS involves inflammatory injury to the endothelium and epithelium of the lung [24]. Previous studies have shown that LPS challenges cause a synergistic induction of systemic inflammation and lung injury [9]. However, the fundamental mechanism underlying the miRNA‐mediated biological function of PMVECs in the context of LPS exposure is still elusive.

The information concerning the roles of miRNAs in LPS‐induced lung injury or ARDS is relatively limited when compared with other diseases, including various cancers, cardiovascular diseases and neurodevelopmental disorders. miR‐494 was reported to be up‐regulated in rat models of sepsis‐associated ARDS. Overexpression of miR‐494 negatively regulated the nuclear erythroid 2‐related factor 2 (NRF2) signaling pathway leading to enhanced inflammatory response and ALI [25]. Similar to miR‐494, the expression of miR‐211 was aberrantly up‐regulated in macrophages of ARDS rat models. Down‐regulation of IL‐10, a target of miR‐211, contributes to the pathological process of ARDS‐associated inflammation [26]. In contrast with miR‐494 and miR‐211, two recent publications reported that miR‐16 and miR‐24 were evidently decreased in lung tissues of LPS‐induced ALI rat models. Interestingly, both miR‐16 and miR‐24 inhibit inflammatory responses in the lung and exert a protective effect against ALI in rats through regulating nucleotide‐binding, oligomerization domain‐like receptor protein 3 (NLRP3) expression with different molecular mechanisms. miR‐16 affects NLRP3 activation through directly targeting Toll‐like receptor 4 (TLR4), and the miR‐16/TLR4 signaling pathway regulates NLRP3 expression via NF‐κB [27], whereas miR‐24 regulates NLRP3 expression by direct targeting NLRP3 [28]. Although these newly identified miRNAs play various and vital roles in LPS‐induced cell injury *in vitro* and *in vivo*, knockdown or restoration of these miRNAs only partially alleviates the symptoms of ALI, suggesting that other miRNAs may be involved in the LPS‐induced ALI/ARDS.

To date, approximately 10 publications related to miR‐642a‐5p can be found in PubMed, highlighting the importance and urgency of revealing the roles of miR‐642a‐5p in various human diseases. miR‐642a‐5p was reported to be associated with survivability in cervical squamous cell carcinoma [29]. miR‐642a‐5p may play a role in the regulation of B cell maturation via targeting DEP domain‐containing protein 6. Furthermore, Sato *et al*. [30] reported that TLR4 is a target of miR‐642a‐5p in THP‐1 cells, but LPS stimulation did not significantly enhance miR‐642a‐5p expression in THP‐1 cells. Dysregulation of miR‐642a‐5p has also been identified in human prostate, bladder, pancreatic and liver cancer [15, 31, 32]. However, the information about the biological roles of miR‐642a‐5p in these cancers is still lacking, and more importantly, whether miR‐642a‐5p plays a role in LPS‐induced ALI/ARDS is obscure.

In this study, we focused on investigating the biological function role of miR‐642a‐5p in the LPS‐stimulated PMVECs. We demonstrated that LPS augmented miR‐642a‐5p expression in PMVECs. LPS has been shown to induce transactivation of miRNA, such as miR‐17‐92, miR‐125b, miR‐30b and miR‐21, through activation of NF‐κB signaling [33]. Whether the same mechanism applied to LPS‐mediated miR‐642a‐5p deserves further investigation. Up‐regulation of miR‐642a‐5p promoted p‐moesin and caspase‐3 activity leading to enhanced permeability and apoptosis of PMVECs. Importantly, we identified that eEF2 is a target of miR‐642a‐5p, and overexpression of eEF2 can partially block the effect of LPS‐ and miR‐642a‐5p‐induced hyperpermeability and apoptosis of PMVECs. eEF2 is known to play a pivotal role in the regulation of autophagy and apoptosis, especially in glucose deprivation condition. Knockdown of eEF2 augments glucose deprivation‐induced caspase‐3 activation and apoptosis [34]. Thus, we believed that LPS induced up‐regulation of miR‐642a‐5p, and the latter down‐regulated eEF2 expression, which subsequently promoted caspase‐3 activation and apoptosis. Notably, Yi *et al*. [35] reported that LPS is capable of inducing PMVEC apoptosis via stimulating Yes‐associated protein translocation from cytoplasm to nucleus to activation of caspase‐3. Here, we presented a novel miR‐642a‐5p‐mediated eEF2/caspase‐3 signaling mechanism in LPS‐induced PMVECs apoptosis.

## Conclusion

Our results demonstrate for the first time that miR‐642a‐5p/eEF2 signaling plays a crucial role in the regulation of the permeability and cell viability of PMVECs with LPS treatment, suggesting miR‐642a‐5p/eEF2 may also be involved in LPS‐induced ALI/ARDS. The findings of this study may provide further insight into understating the molecular mechanism of LPS‐induced ALI/ARDS.

## Conflict of interest

The authors declare no conflict of interest.

## Author contributions

LF, GS and QY conceived and designed the experiments; LF performed the experiments; QY analyzed and interpreted the data and contributed reagents, materials, analysis tools or data; GS wrote the paper.

## Data Availability

Data are available from the corresponding author upon reasonable request.
